# TIE2-expressing monocytes/macrophages regulate revascularization of the ischemic limb

**DOI:** 10.1002/emmm.201302752

**Published:** 2013-05-07

**Authors:** Ashish S Patel, Alberto Smith, Silvia Nucera, Daniela Biziato, Prakash Saha, Rizwan Q Attia, Julia Humphries, Katherine Mattock, Steven P Grover, Oliver T Lyons, Luca G Guidotti, Richard Siow, Aleksandar Ivetic, Stuart Egginton, Matthew Waltham, Luigi Naldini, Michele De Palma, Bijan Modarai

**Affiliations:** 1 Academic Department of Surgery, Cardiovascular Division, King's College London, BHF Centre of Excellence and the Biomedical Research Centre at Guy's & St Thomas' NHS Foundation Trust and King's College LondonUK; 2 Angiogenesis and Tumor Targeting Unit, and HSR-TIGET, San Raffaele Scientific InstituteMilan, Italy; 3 Vita-Salute UniversityMilan, Italy; 4 Swiss Institute for Experimental Cancer Research (ISREC), School of Life Sciences, Swiss Federal Institute of Technology Lausanne (EPFL)Lausanne, Switzerland; 5 Immunopathogenesis of Liver Infections Unit, San Raffaele Scientific InstituteMilan, Italy; 6 Vascular Biology Group, Cardiovascular Division, King's College LondonUK; 7 Membrane/Cytoskeletal Signalling Group, Cardiovascular Division, King's College LondonUK; 8 School of Biomedical Sciences, Faculty of Biological Sciences, University of LeedsLeeds, UK

**Keywords:** angiogenesis, limb ischemia, macrophages, monocytes, TIE2

## Abstract

A third of patients with critical limb ischemia (CLI) will eventually require limb amputation. Therapeutic neovascularization using unselected mononuclear cells to salvage ischemic limbs has produced modest results. The TIE2-expressing monocytes/macrophages (TEMs) are a myeloid cell subset known to be highly angiogenic in tumours. This study aimed to examine the kinetics of TEMs in patients with CLI and whether these cells promote neovascularization of the ischemic limb. Here we show that there are 10-fold more circulating TEMs in CLI patients, and removal of ischemia reduces their numbers to normal levels. TEM numbers in ischemic muscle are two-fold greater than normoxic muscle from the same patient. TEMs from patients with CLI display greater proangiogenic activity than TIE2-negative monocytes *in vitro*. Using a mouse model of hindlimb ischemia, lentiviral-based *Tie2* knockdown in TEMs impaired recovery from ischemia, whereas delivery of mouse macrophages overexpressing TIE2, or human TEMs isolated from CLI patients, rescued limb ischemia. These data suggest that enhancing TEM recruitment to the ischemic muscle may have the potential to improve limb neovascularization in CLI patients.

→See accompanying articles http://dx.doi.org/10.1002/emmm.201302695 and http://dx.doi.org/10.1002/emmm.201302794

## INTRODUCTION

Peripheral arterial occlusive disease (PAOD) affects one in five individuals over the age of 75 (Berger and Hiatt, 2012). The spectrum of PAOD ranges from intermittent claudication to critical limb ischemia (CLI). The latter results from severe restriction of blood flow and manifests as constant and intractable pain, often with ulceration or gangrene of the limb (Norgren et al, 2007; Varu et al, 2010). Conventional interventions for this condition include arterial bypass surgery or endovascular therapies such as balloon angioplasty and stenting (Norgren et al, 2007; Varu et al, 2010). A significant proportion of patients with CLI are, however, not amenable to these treatments and up to a fifth will require amputation of the limb within a year (Marston et al, 2006). The quality of life of patients with CLI is similar to those with terminal cancer (Albers et al, 1992) underlining the need for more effective revascularization strategies in these patients.

Therapeutic neovascularization has been hailed as a promising treatment for patients who cannot be revascularized using conventional methods, but the majority of clinical studies that have used angiogenic growth factors alone have reported limited efficacy (Belch et al, 2011; Lederman et al, 2002; Rajagopalan et al, 2003). This has stimulated investigations into the utility of cell-based therapy as a means of sustained production of the complex mixture of growth factors required for robust, efficacious revascularization, but results obtained after injection of unselected bone marrow (BM) or peripheral blood-derived mononuclear cell isolates have also been equivocal (Fadini et al, 2010; Moazzami et al, 2011). This may have resulted from ‘dilution’ of the delivered angiogenic cells in these mixed cell populations. Identification and selective delivery of a specific, potent angiogenic cell population may, therefore, be the key to developing more efficacious treatments (Losordo and Dimmeler, 2004).

In pre-clinical models, there is strong evidence to show that TIE2-expressing monocytes/macrophages (TEMs) support angiogenesis in tumours and remodelling tissues (Capobianco et al, 2011; Coffelt et al, 2010; De Palma et al, 2005; Fantin et al, 2010; He et al, 2012; Mazzieri et al, 2011; Modarai et al, 2005; Pucci et al, 2009), but there is a paucity of data linking this cell type to pathologies in patients. Work in animal models suggests that their role is to provide paracrine support for angiogenesis by cross-talking with, or bridging endothelial cells to aid tip-cell fusion (Fantin et al, 2010; Mazzieri et al, 2011). Specific depletion of TEMs (Capobianco et al, 2011; De Palma et al, 2005) or conditional *Tie2* knockdown in these cells (Mazzieri et al, 2011) inhibits tumour angiogenesis, which supports the notion that TEMs represent an important angiogenic drive in these pathological tissues. A recent clinical study also showed that circulating TEMs are increased in hepatocellular carcinoma patients and preferentially localize in the perivascular areas of the tumour tissue (Matsubara et al, 2013).

Here, we investigate whether TEMs have a role in the revascularization of the ischemic limb by: (i) determining whether TEMs are present in the circulation and ischemic muscle of CLI patients; (ii) examining the functional relationship between TIE2 expression on monocytes and their proangiogenic activity *in vitro* and in the ischemic limb *in vivo*.

## RESULTS

### TEMs are increased in patients with CLI and are found within ischemic muscle

**Figure 1:**
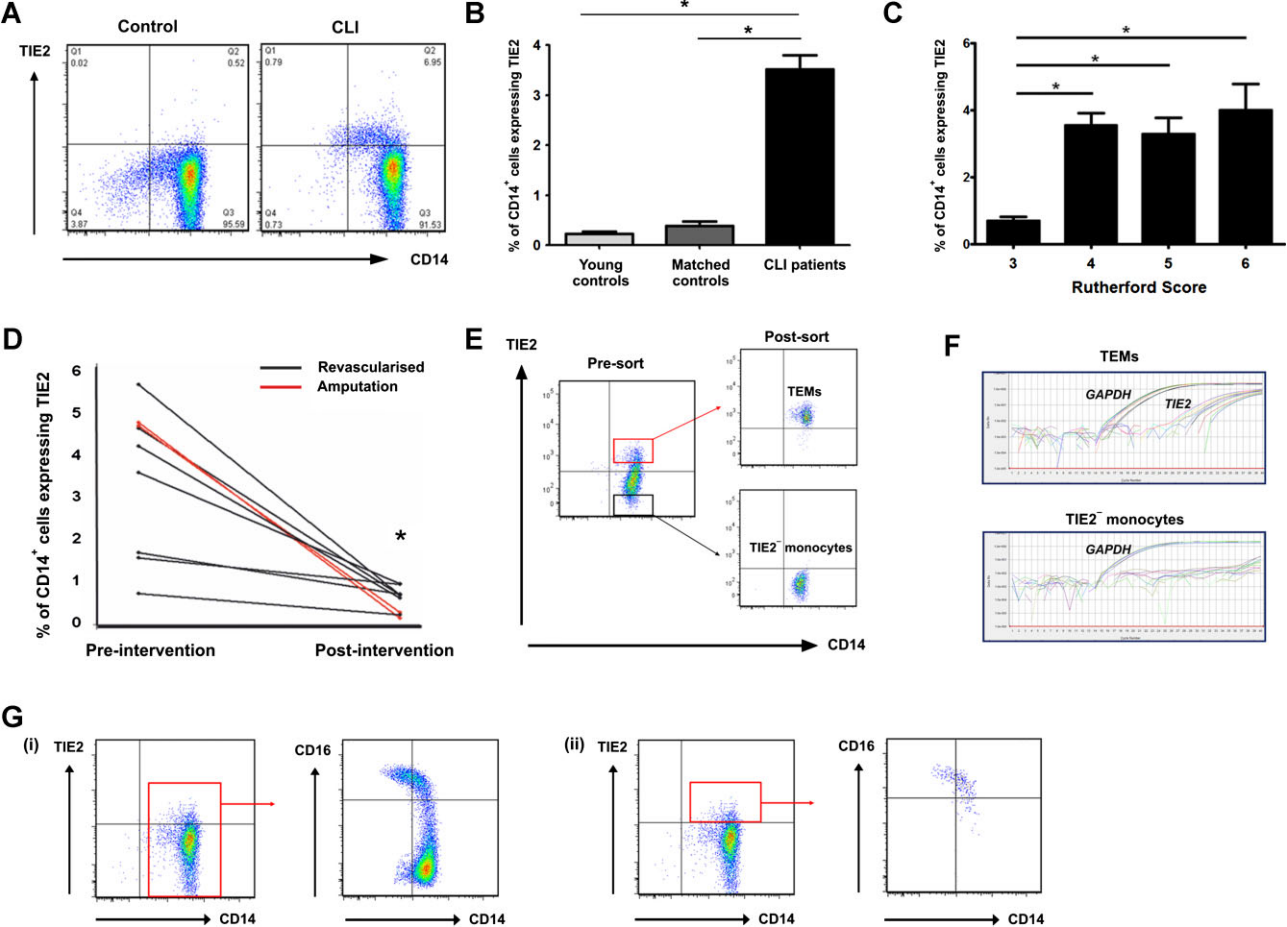
Changes in circulating and muscle resident TEMs in response to CLI Representative flow cytometric dot plot of circulating TEMs (top right hand gates) in a patient with CLI (right) compared with an age-matched control (left) showing a higher proportion of monocytes that express TIE2 in the patient.CLI patients (*n* = 40) have a higher proportion of monocytes expressing TIE2 compared with young (*n* = 20) and age-matched (*n* = 20) controls (3.52 ± 0.28% *vs*. 0.23 ± 0.04% and 0.39 ± 0.09% respectively). *p < 0.0001 by two-tailed Mann-Whitney U test. Data are mean ± SEM.Circulating TEMs are significantly higher in CLI patients (*i.e*. those with ischemic rest pain or gangrene; Rutherford Score 4, 5 and 6) compared with patients with intermittent claudication (Rutherford Score 3, *p* < 0.001 by one-way ANOVA). *p < 0.05 by post-hoc Bonferroni for Rutherford 3 *versus* 4, 5 and 6.Graph shows a significant fall in circulating TEMs after removal of the ischemic stimulus in CLI patients by either surgical revascularization (black lines) or amputation (red lines). *p < 0.005 by two-tailed paired *t*-test.FACS-sorting of TEMs (top gate, red) and TIE2^−^ monocytes (bottom gate, black). Post-sort purity check (right dot plots) show high purities, 94.5% ± 0.8 for TEMs (*n* = 5 samples).RT-PCR traces showing that expression of *TIE2* is present in TEM samples after 25 cycles but is absent in TIE2^−^ monocytes. *n* = 8 CLI patients, TIE2^+^ and TIE2^−^ samples analysed in triplicate.(i) Gating of the whole monocyte population (red gate) for phenotyping according to CD14 and CD16 expression shows the typical distribution of classical (CD14^++^CD16^−^ bottom right quandrant), intermediate (CD14^++^CD16^+^, top right quadrant) and non-classical (CD14^+^CD16^+^, top left quadrant) monocytes. (ii) Gating of TEMs (red gate) for phenotyping according to CD14 and CD16 expression shows that the majority of these cells express CD16 and are, therefore, found within either the intermediate or non-classical subset.

We compared TIE2 expression in circulating monocytes from patients with CLI and matched controls using flow cytometry. The demographics of the subjects recruited into this study are listed in Table 1. Patients with CLI were well matched with controls for age, sex, smoking history and the co-morbidities associated with peripheral arterial disease, including hypertension, hyperlipidemia, diabetes and ischemic heart disease (*p* > 0.05 by Fisher's exact test for each). We found that the proportion of circulating CD14^+^ monocytes that expressed TIE2 was 9-fold and 15-fold greater in CLI patients compared with age-matched and young controls, respectively (*p* < 0.0001, Fig 1A and B, and Supporting Information Fig S1). Circulating TEM numbers were significantly higher in CLI patients (*i.e*. those with ischemic rest pain or gangrene; Rutherford Score 4, 5 and 6) compared with patients with intermittent claudication [Rutherford Score 3, *p* < 0.001 by one-way analysis of variance (ANOVA), *p* < 0.05 by post-hoc Bonferroni for Rutherford 3 *vs*. 4, 5 and 6, Fig 1C].

**Table 1:** Demographics of CLI patients, age-matched and young controls

Characteristic	CLI (*n* = 40)	Age-matched controls (*n* = 20)	Young controls (*n* = 20)
Age (range)	73 (59–91)	72 (58–88)	35 (21–38)
Male	23 (66%)	13 (65%)	21 (60%)
Positive smoking history	34 (85%)	15 (75%)	7 (35%)
Hypertension	31 (78%)	15 (75%)	0
Hyperlipidemia	25 (63%)	11 (55%)	0
Diabetes	5 (13%)	3 (15%)	0
Ischemic heart disease	9 (23%)	7 (35%)	0
Rutherford Score			
4	18 (45%)		
5	17 (43%)		
6	5 (12%)		
Mean ABPI ± sem	0.4 ± 0.09		

No significant difference in demographics between the two groups (CLI *vs*. age-matched controls, *p* > 0.05 by Fisher's exact test). Rutherford scores: 4: ischemic rest pain; 5: rest pain with minor tissue loss; 6: rest pain with major tissue loss. ABPI: ankle:brachial artery pressure index (a measure of restriction to blood flow in peripheral arterial disease where a ratio of 1.0 suggests normal flow).

To examine whether this rise in TEMs in CLI patients was a specific response to tissue ischemia, circulating TEMs were measured in a group of CLI patients prior to and 12 weeks after successful removal of the ischemic stimulus by either revascularization or amputation of the affected limb. Circulating TEM numbers in these patients fell to levels seen in controls (*p* < 0.004, Fig 1D).

Expression of the *TIE2* transcript in TEMs was confirmed using quantitative PCR after fluorescence-activated cell sorting (FACS) of TIE2^+^ and TIE2^−^ monocytes from blood (Fig 1E and F). Monocytes were further separated according to their expression of CD14 and CD16 into the three main monocyte subsets previously described; classical (CD14^++^CD16^−^), non-classical (CD14^+^CD16^+^) and intermediate (CD14^++^CD16^+^) (Geissmann et al, 2010). The majority of TEMs (82 ± 5%) fell within the CD16^+^ monocyte population, suggesting that TIE2 expression on monocytes is associated with a non-classical/intermediate monocyte phenotype (Fig 1G).

**Figure 2:**
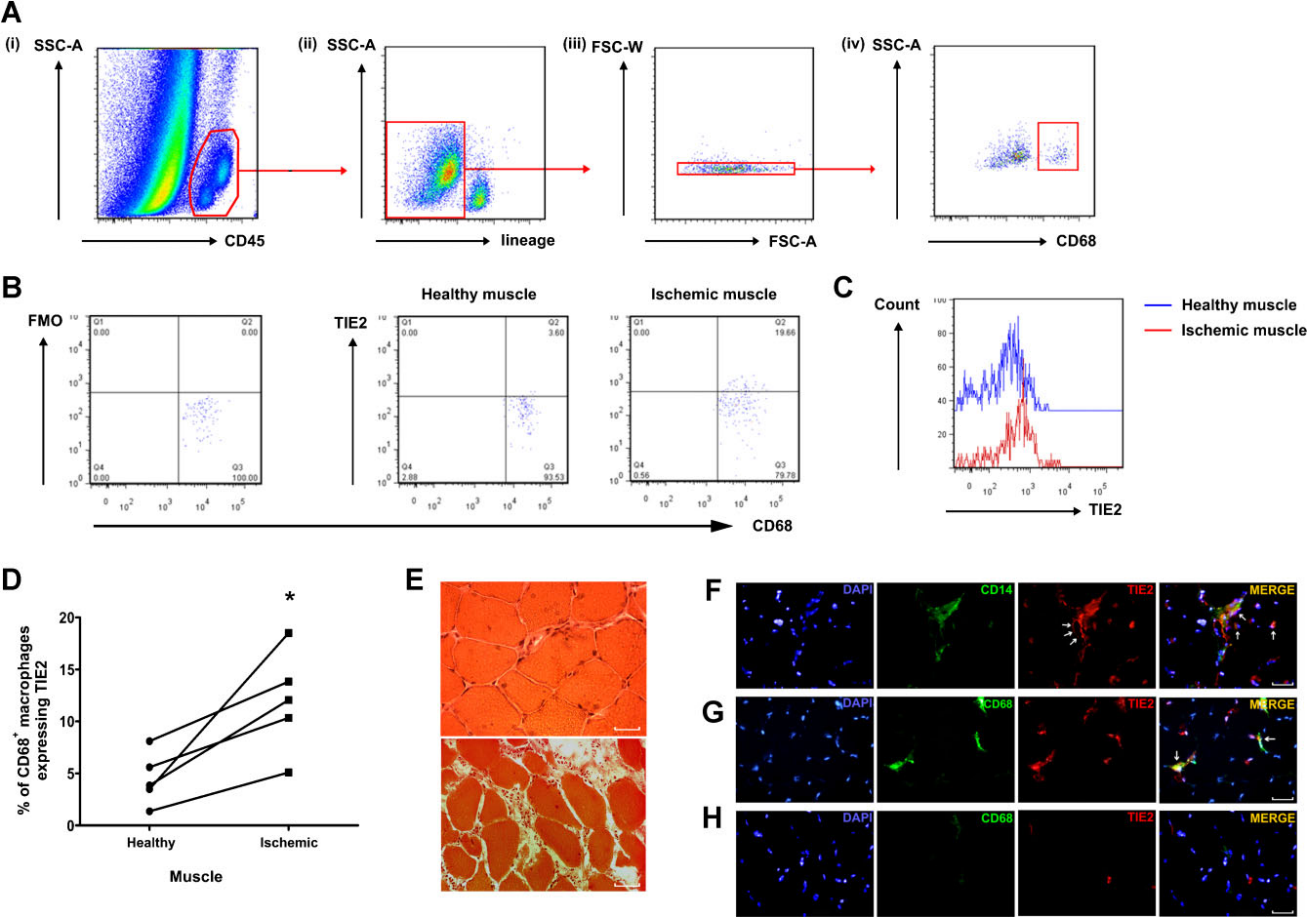
Quantification of TIE2^+^ macrophages in human muscle specimens Muscle specimens were enzymatically digested and analysed by flow cytometry. Gating (red gates) of CD45 positive cells (i) followed by exclusion of lineage (CD19, CD56, CD3) positive cells (ii), exclusion of doublets (iii) and selection of CD68^+^ macrophages (iv).Gate for TIE2 expression set according to staining with FMO sample (left). Example TIE2 staining of cells from healthy muscle (middle) and ischemic muscle (right) showing a higher proportion of TIE2^+^ macrophages in the ischemic compared with normal tissue.Histogram (gated on CD68^+^ macrophages) showing higher expression of TIE2 in macrophages from ischemic (red) compared with healthy (blue) muscle.Flow cytometry analysis of digested muscle specimens shows higher proportion of CD68^+^ macrophages expressing TIE2 in distal ischemic muscle compared with proximal healthy muscle biopsies from CLI patients (11.3 ± 2.2% *vs*. 4.5 ± 1.3%, respectively). *p < 0.05 by paired *t*-test.H&E sections of normoxic (top) muscle compared with ischemic (bottom) muscle which shows loss of the normal muscle architecture and cellular infiltrate. Scale bars represent 50 µm.Immunofluorescence stains of a section of ischemic muscle showing nucleated cells (blue) expressing CD14 (green) and TIE2 (red) near a blood vessel lined with TIE2-expressing endothelial cells (arrows). Merged image shows TEMs (orange, arrows).Section of ischemic muscle showing nucleated cells (blue) expressing CD68 (green) and TIE2 (red). Merged image shows macrophages expressing TIE2 (orange, arrows).Section of healthy muscle showing less frequent nucleated cells (blue) expressing CD68 (green) and TIE2 (red). TIE2-expresssing macrophages are not readily seen. Scale bars represent 50 µm.

We also located and quantified TEMs in distal (ischemic) and proximal (normoxic) muscle biopsies from the limbs of CLI patients by immunofluorescence staining of frozen sections or flow cytometric analysis of enzymatically-digested specimens. Greater numbers of TIE2^+^ macrophages were present in ischemic (11.3 ± 2.2%) compared with normoxic muscle from the same individuals (4.5 ± 1.3%. *p* < 0.05, Fig 2A–H).

### TEMs have proangiogenic activity and respond to angiopoietin stimulation

**Figure 3:**
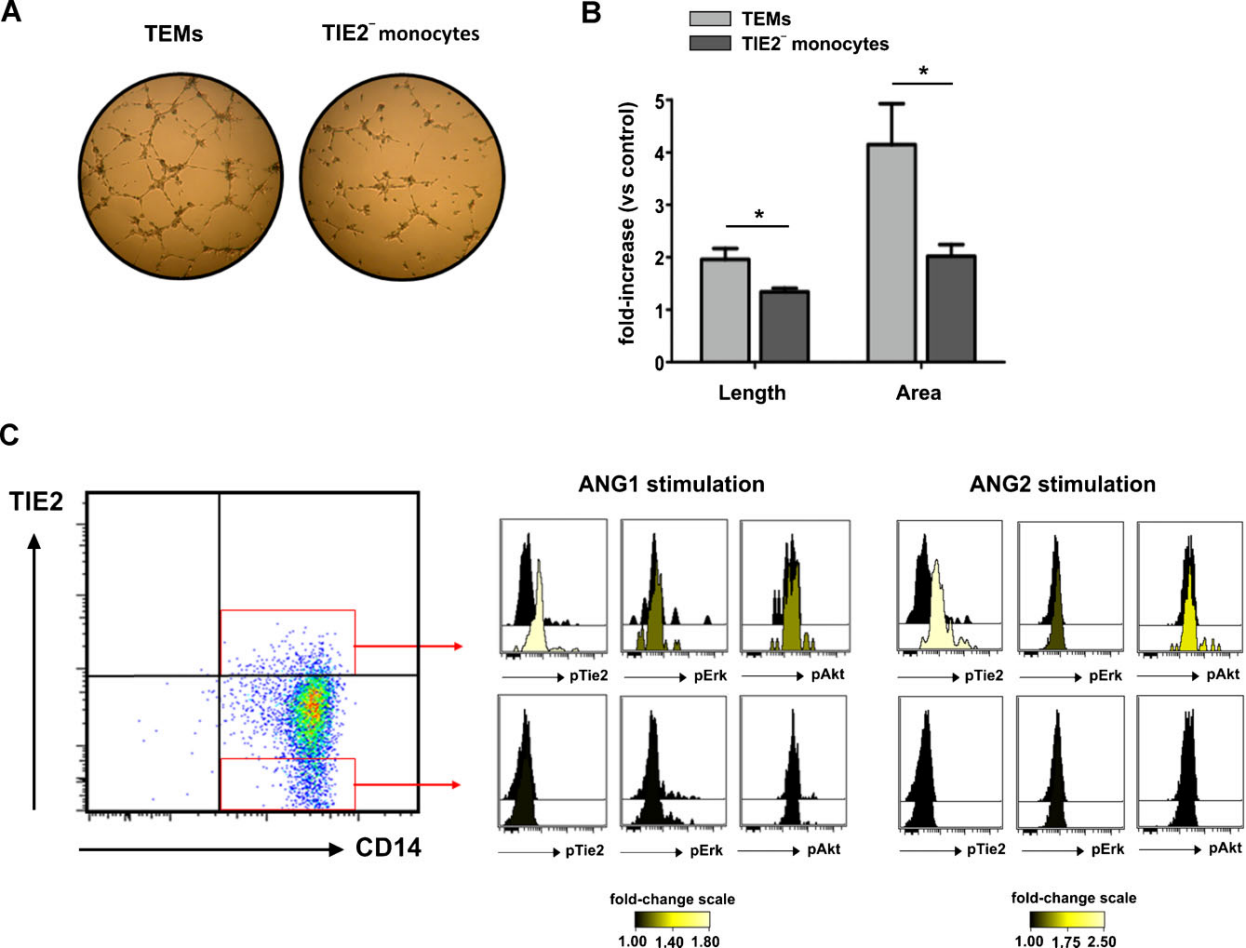
Proangiogenic activity of TEMs Typical example of tubules formed following co-culture of HUVECs with TEMs from a CLI patient (left) compared with TIE2^−^ monocytes from the same individual (right).Overall, there is greater tubule formation (for both tubule length and area) when HUVECs are co-cultured with TEMs compared with TIE2^−^ monocytes. Each assay performed in triplicate; cells obtained from five CLI patients and five matched-controls. Fold-change in tubule formation was calculated by comparing tubule growth with control (HUVECs alone) tubules in the same assay. Values shown are mean ± SEM. *p < 0.05 by 2-tailed *t*-test.Histograms show phosphorylation of TIE2 and downstream ERK and AKT signalling in TEMs (upper gate in red) and TIE2^−^ monocytes (lower gate in red) in unstimulated samples (upper histograms) compared with ANG1 and ANG2-stimulated samples (lower histograms). Stimulation with ANG1 and ANG2 induces phosphorylation of TIE2, ERK and AKT in TEMs but not in TIE2^−^ monocytes. Phosphorylation measured as fold-change in median-fluorescence intensity of staining. Representative histograms, *n* = 5 for each, performed in duplicate.

TEMs are known to have proangiogenic functions both *in vitro* and *in vivo* (Coffelt et al, 2010; De Palma et al, 2005) but the activity of TEMs isolated from aged CLI patients with multiple co-morbidities has not previously been investigated. TEMs isolated from the blood of CLI patients and co-cultured with HUVECs on Matrigel exhibited a greater capacity to enhance HUVEC tubule formation compared with TIE2^−^ monocytes from the same individuals (*p* < 0.05, Fig 3A and B). Having identified differences in the numbers and proangiogenic activity of circulating and muscle-resident TEMs between CLI and controls, we next measured a panel of circulating angiogenic and proinflammatory factors in the plasma of CLI patients and compared this with controls (Table 2). The levels of angiopoietin-2 (ANG2, a TIE2 ligand), vascular endothelial growth factor (VEGF) and soluble TIE2 (sTIE2) were significantly raised in CLI patients compared with matched controls (*p* < 0.05 for all). Levels of angiopoietin-1 (ANG1) were also twofold higher in CLI patients compared with controls. ANG1 and ANG2 phosphorylate the TIE2 receptor in endothelial cells and ANG2 in particular regulates proangiogenic gene expression in TEMs (Coffelt et al, 2010). We, therefore, stimulated peripheral blood mononuclear cells (PBMCs) from CLI patients with both ANG1 and ANG2 and used intracellular flow cytometric analysis to measure downstream signalling in order to determine whether the TIE2 receptor is functional in TEMs from patients with CLI. Both angiopoietins phosphorylated the TIE2 receptor on these cells, resulting in activation of the downstream phosphokinases, ERK and AKT (Fig 3C).

**Table 2:** Measurement of circulating factors in the plasma of CLI patients *versus* matched controls

	Age-matched controls (pg/mL)	Critical limb ischemia (pg/mL)	*p*-value
ANG1	3771 ± 1418	7442 ± 2463	ns
ANG2	1973 ± 247	4354 ± 661	<0.05
FGF	29 ± 10	40 ± 19	ns
PLGF	11 ± 4	13 ± 4	ns
VEGFR1	91 ± 28	68 ± 9	ns
VEGFR2	6824 ± 1038	6557 ± 1008	ns
VEGF	63 ± 21	297 ± 117	<0.05
sTIE2	19,500 ± 1400	25,900 ± 1900	<0.05
PECAM-1	49,763 ± 3312	68,571 ± 8820	<0.05
VCAM-1	325,816 ± 57,555	403,462 ± 52,218	ns
IL-4	1.8 ± 0.1	1.5 ± 0.1	ns
IL-10	11.9 ± 6.4	2.5 ± 0.6	ns
IL-6	17 ± 6	79 ± 29	<0.05
TNF-α	4.4 ± 0.4	6.7 ± 2.0	ns
MCP-1	353 ± 88	295 ± 53	ns
MCSF	14 ± 1	39 ± 11	<0.05
GMCSF	3.59 ± 1.3	8.79 ± 4.3	ns
SDF-1	334 ± 80	387 ± 68	ns

Levels of ANG2, VEGF, sTIE2, PECAM-1, IL-6 and MCSF were significantly higher in CLI. *n* = 10 subjects per group. *p* < 0.05 by Mann-Whitney U test. ns: not statistically significant.

### Characterization of TEMs in a mouse model of hindlimb ischemia (HLI)

**Figure 4:**
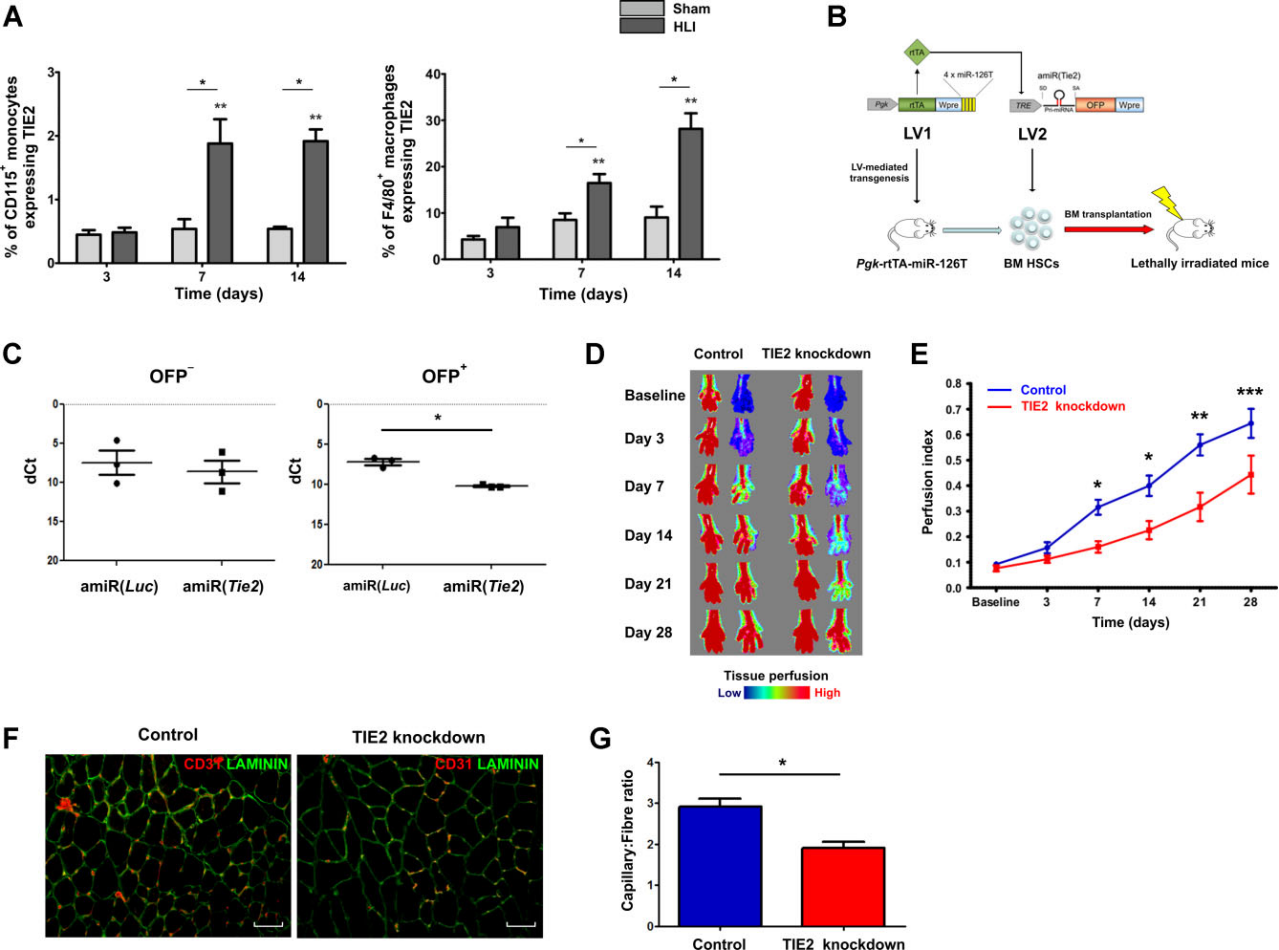
TIE2-expressing monocytes/macrophages are upregulated following HLI; silencing their expression of *Tie2* inhibits revascularization Significant increase in circulating TEMs and muscle-resident TIE2^+^ macrophages following HLI at day 7 and day 14. *p < 0.05 *versus* sham for same timepoint; **p < 0.05 *versus* HLI at day 3 by one-way ANOVA. *n* = 5–7 mice per group.Schematic diagram of double-lentiviral siRNA-mediated knockdown of *Tie2* expression.RT-PCR analysis to measure *Tie2* expression in transduced (OFP^+^) and untransduced (OFP^−^) myeloid cells isolated from the spleens of both amiR(*Tie2*) and amiR(*Luc*) mice (4 weeks after HLI induction; *n* = 9 mice/group). The plots show the dCt mean values for each sample. Significant reduction of *Tie2* expression was found in the amiR(*Tie2*) group compared with the amiR(*Luc*) group for OFP^+^ (right) and not OFP^−^ (left) myeloid cells. *p < 0.002 by Mann-Whitney U test. *n* = 3 biological samples per group; each sample has been analysed in duplicate and represents a pool of cells from 3 mice. Error bars represent SEM.Laser Doppler images of paw perfusion in representative control (left) and TIE2 knockdown (right) mice following unilateral HLI. Images show faster recovery of paw perfusion in the controls compared with the TIE2 knockdown mice.Perfusion index graph shows a significant reduction in paw perfusion following knockdown of TIE2 in TEMs (red line) compared with control mice (blue line); *p* < 0.0001 by two-way ANOVA. Post-hoc Bonferroni tests: *p < 0.05; **p < 0.001; ***p < 0.01. *n* = 8–10 mice per group.Mouse gastrocnemius muscle stained for CD31 (red) and laminin (green) and used to calculate capillary:fibre (C:F) ratio (outline of muscle fibres appear green and capillaries, that stain for both, appear orange). The C:F ratio is reduced in muscle from a *Tie2* knockdown mouse compared with a control.Overall, a significantly lower C:F ratio in the muscle of TIE2 knockdown mice compared with control mice (*n* = 5 mice/group). *p < 0.001. Scale bars represent 100 µm.

We next determined whether the TEM kinetics we had observed in patients with CLI would be recapitulated in a mouse model of severe HLI that simulates CLI in man. In this model the proximal and distal femoral artery (and its branches) are ligated and the intervening segment is excised, causing marked hypoperfusion of the lower leg and foot, resulting in gangrene of the toes (Supporting Information Fig S2A). Flow cytometry (Supporting Information Fig S2B-D) showed a 3.5-fold increase in the proportion of circulating TEMs (defined as TIE2^+^CD11b^+^CD115^+^ monocytes) after induction of HLI at 7 days (1.88 ± 0.38% *vs*. 0.52 ± 0.16%, *p* < 0.001 by post-hoc Bonferroni) and 14 days (1.92 ± 0.19% *vs*. 0.54 ± 0.03%, *p* < 0.001 by post-hoc Bonferroni, for HLI and sham, respectively). This mirrored a twofold increase in the numbers of TIE2^+^ tissue-resident macrophages (CD45^+^CD11b^+^F4/80^+^ cells) in ischemic, compared with normoxic, muscle at 7 days (16.46 ± 1.92% *vs*. 8.52 ± 1.41%, *p* < 0.05 by post-hoc Bonferroni) and a threefold increase at 14 days (28.16 ± 3.35% *vs*. 9.03 ± 2.35%, *p* < 0.001 by post-hoc Bonferroni, Fig 4A); a result that was strikingly similar to the cellular response seen in CLI patients.

### Silencing of *Tie2* in circulating TEMs impairs revascularization of the ischemic murine hindlimb

Selective elimination of TEMs in tumour-bearing mice impairs angiogenesis and slows tumour growth (De Palma et al, 2005). Furthermore, the expression of TIE2 on these cells has been shown to be important for their proangiogenic function in tumours (Mazzieri et al, 2011). We, therefore, investigated the effect of silencing monocyte TIE2 expression on resolution of HLI in the mouse to determine whether TIE2 expression on TEMs is also important for their role in revascularizing the ischemic limb. We used an inducible lentiviral vector (LV)-based platform previously described (Mazzieri et al, 2011) to knockdown *Tie2* in TEMs (Fig 4B). Briefly, we replaced the stem sequence of microRNA-223 with small interfering RNA (siRNA) sequences targeting *Tie2* to generate the artificial microRNA, amiR(*Tie2*); we also generated a control amiR targeting Luciferase, termed amiR(*Luc*). These LV constructs, expressing the marker gene orange fluorescent protein (OFP), were transduced *ex vivo* into BM-derived hematopoietic stem/progenitor cells (HS/PC) obtained from transgenic FVB/Pgk-rtTA-miR-126T mice, generated by LV-mediated transgenesis (Mazzieri et al, 2011). Transduced/transgenic cells were used to reconstitute the BM of lethally irradiated FVB mice. In these mice, *Tie2* expression can be conditionally silenced specifically in mature hematopoietic cells by suppressing expression of the rtTA in HS/PCs through endogenous miR-126 activity. Effective *Tie2* silencing was confirmed by showing that the *Tie2* transcript levels were significantly down-regulated in FACS-sorted OFP^+^ myeloid cells (*vs*. OFP^−^ cells) obtained from doxycycline-treated amiR(*Tie2*) but not amiR(*Luc*) mice (Fig 4C and Supporting Information Fig S3). Remarkably, doxycycline-induced silencing of *Tie2* in TEMs inhibited the endogenous ‘rebound’ angiogenic response that normally recovers blood perfusion to the ischemic limb over a 28 day period in this model (Fig 4D and E, *p* < 0.0001 by two-way ANOVA). Indeed, laser Doppler imaging showed that, at day 7 post-ischemia, there was a significantly lower paw perfusion index in mice in which *Tie2* was silenced in TEMs (*p* < 0.05 by post-hoc Bonferroni test), and this difference persisted throughout the course of the study up to day 28 (*p* < 0.01). This also corresponded with significantly reduced capillary:fibre ratio in gastrocnemius muscle tissue harvested at the end of the experiment and analysed histologically in amiR(*Tie2*) compared with control mice (Fig 4F and G, *p* < 0.001 by Mann-Whitney U test). These findings indicate that the TIE2 receptor functionally contributes to the proangiogenic activity of TEMs in the ischemic skeletal muscle and that these cells have an important role in revascularization of the limb.

### Delivery of TEMs into the ischemic hindlimb accelerates revascularization and improves limb salvage

**Figure 5:**
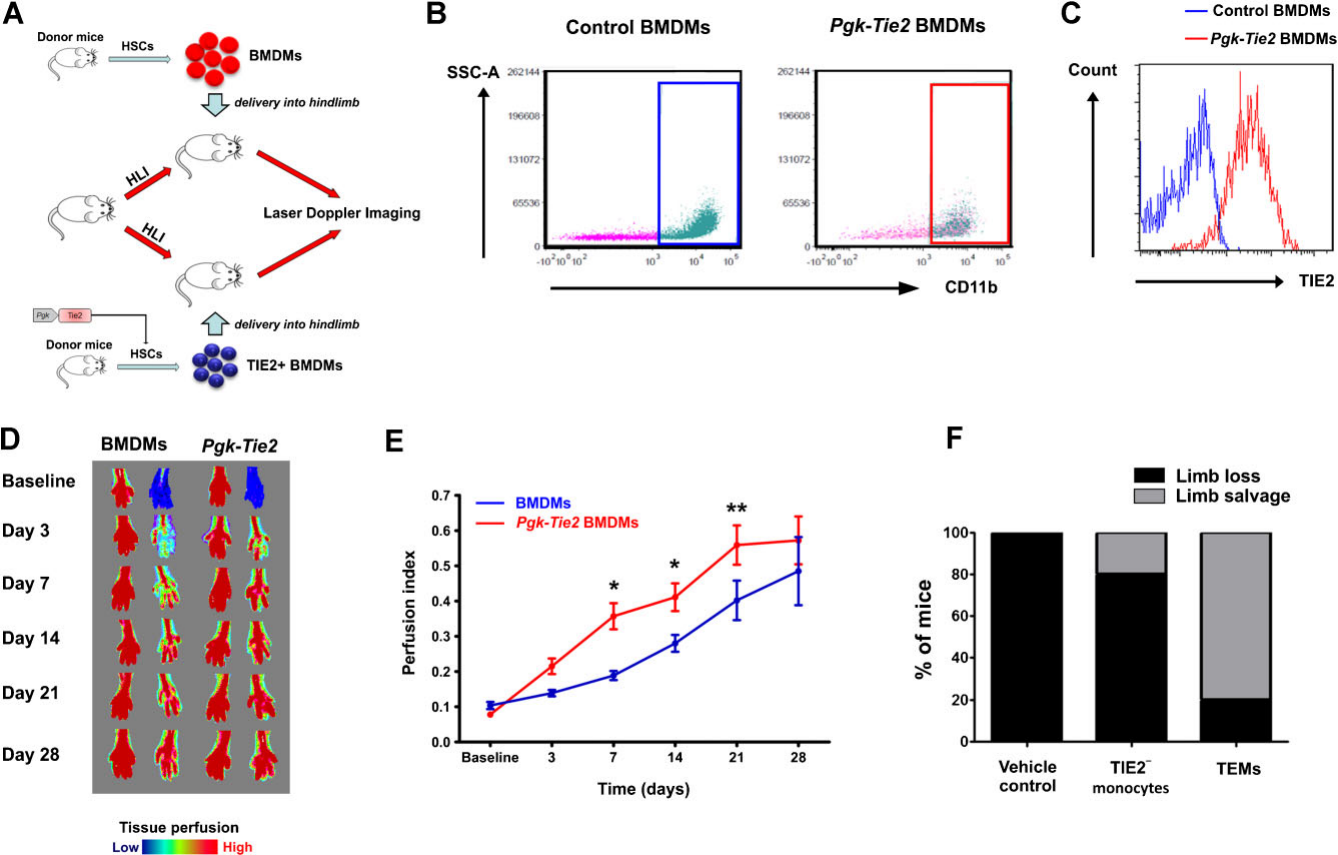
Delivery of (i) murine bone marrow derived TIE2^+^ macrophages and (ii) TEMs from CLI patients into the ischemic hindlimb accelerates revascularization Schematic diagram showing generation of TIE2^+^ BMDMs via LV-mediated transduction of *Pgk-Tie2* lentivirus and delivery of these cells into the ischemic hindlimb 24 h following induction of HLI. Limb perfusion was then imaged at days 3, 7, 14, 21 and 28.CD11b-expression of cultured HSCs following *Pgk*-*Tie2* transduction (red gate) *versus* control BMDMs (blue gate).Histogram shows marked upregulation of TIE2 expression on *Pgk*-*Tie2* BMDMs (red) compared with control cells (blue).Laser Doppler images of paw perfusion in representative ischemic hindlimbs injected with control BMDMs (left) and *Pgk*-*Tie2* BMDMs (right) showing accelerated recovery of paw perfusion in the *Pgk*-*Tie2* treated group.Paw perfusion index graph shows significantly faster paw perfusion recovery following delivery of *Pgk*-*Tie2* BMDMs (red) compared with control BMDMs (blue line); *p* < 0.0001 by two-way ANOVA. Post-hoc Bonferroni tests: *p < 0.05; **p < 0.01. *n* = 8–10 mice per group.Increased salvage of ischemic hindlimbs of nude, athymic mice following delivery of human TEMs (80%, *n* *=* 4/5) compared with TIE2^−^ monocytes (20%, *n* = 1/5) and vehicle control (0%, *n* = 0/5).

We then investigated the therapeutic potential of TEMs in the HLI model by enforcing the expression of TIE2 on BM-derived macrophages (BMDMs) using a *Pgk*-*Tie2* LV (Fig 5A). The enforced expression of TIE2 in murine CD11b+ BMDMs was confirmed by flow cytometry (Fig 5B and C). TIE2-expressing or control BMDMs (5 × 10^5^ per group) were injected into the adductor muscle of the ischemic hindlimb and revascularization was measured using laser Doppler. Delivery of TIE2-expressing BMDMs enhanced revascularization of the ischemic limb compared with wild-type BMDMs (Fig 5D and E). We then investigated whether TEMs isolated from CLI patients have a similar capacity to stimulate revascularization of the ischemic hindlimb. Injection of TEMs (5 × 10^5^ per group) from CLI patients into the ischemic hindlimbs of nude, athymic mice similarly protected against limb loss compared with animals injected with TIE2^−^ monocytes isolated from the same patients (Fig 5F). The hindlimb salvage rate after injection of TEMs from CLI patients was 80% compared with 20 and 0% after delivery of TIE2^−^ monocytes and vehicle control, respectively.

## DISCUSSION

TIE2-expressing monocytes are thought to be important for the development of tumour blood vessels and have been highlighted as a potential target to inhibit tumour angiogenesis and growth (De Palma et al, 2007). In this study, we show that while circulating TEM numbers are over 10-fold higher in patients with CLI than in matched controls, the difference in muscle, although significant, is less pronounced. Poor limb perfusion following the onset of critical ischemia may indeed limit TEM recruitment to the ischemic limb, and possibly explain why TEMs do not obviously rescue the ischemic limb in CLI patients. Poor limb perfusion could also account for the lack of muscle revascularization in spite of the increased levels of circulating angiogenic factors (such as VEGF and ANG2) in patients with CLI. Furthermore, it is also possible that recruited TEMs do not survive in the hostile environment of the ischemic muscle shortly after recruitment. It is important to note that the increase in circulating TEM numbers was only associated with the presence of critical ischemia rather than with its severity (assessed by Rutherford category). There were no other clinical correlates (such as diabetes or age) with circulating TEM numbers.

The data from the present study suggest that TEMs fall into both CD16^+^ monocyte subsets identified based on the intensity of expression of CD14, *i.e*., non-classical CD14^+^CD16^+^ and intermediate CD14^++^CD16^+^ cells. The intermediate monocyte subset was shown to differentially express high levels of *TIE2* as well as several other proangiogenic genes, including endoglin (*EDG1*) and *VEGFR2* (Zawada et al, 2011).

We also provide *in vivo* evidence that TEMs have a role in regulating neovascularization in limb ischemia. Monocytes are the only sizable mononuclear cell population that express TIE2 in the circulation, and the selective elimination of TEMs in tumour-bearing mice impairs angiogenesis and slows tumour growth (De Palma et al, 2005). Silencing the expression of TIE2 on TEMs impaired the restoration of blood flow to the ischemic hindlimb and this impairment persisted throughout the course of the experiment, suggesting that TEMs have an important role in revascularization of ischemic tissue. Direct delivery of murine BMDMs overexpressing TIE2 into the ischemic hindlimb accelerated the resolution of ischemia (improved perfusion was noted as early as 48 h after delivery of these cells), further supporting a role for TEMs in muscle neovascularization. TEMs isolated from CLI patients also prevented the onset of gangrene and auto-amputation after induction of HLI in nude mice. These data suggest that TEMs have the capacity to promote neovascularization *in vivo* and support the notion that the lack of an effect in CLI patients, in the face of large circulating TEM numbers, may be as a result of poor recruitment to the muscle.

The angiogenic hypoxia-inducible factor (HIF) pathway is activated in ischemic muscle of patients with acute-on-chronic ischemia (Tuomisto et al, 2004). This results in transcriptional upregulation of genes containing hypoxia responsive elements, including VEGF and tumour necrosis factor α (TNF-α), which promote release of ANG2 by endothelial cells within the ischemic muscle (Tressel et al, 2008). It is possible, therefore, that the endothelium is the source of the increased ANG2 levels we, and others, have measured in the blood (and muscle) of patients with CLI (Brandao et al, 2011; Findley et al, 2008). We now show that stimulation of TEMs from CLI patients with ANG2 (as well as ANG1) induces phosphorylation of the TIE2 receptor and activates downstream signalling. These data suggest that circulating TEMs have marked proangiogenic activity and that their ligands, especially ANG2 which is increased in the circulation of CLI patients, may regulate activation of the TIE2 receptor and downstream signalling *in vivo*. The raised levels of circulating ANG2 in CLI patients could enhance the angiogenic activity of TEMs whilst they are in the circulation before they infiltrate the ischemic muscle as shown by Hamm et al (2013) and others (Coffelt et al, 2010). TIE2-expressing monocytes do not express the chemokine (C-C motif) receptor 2 (CCR2) and, rather than responding to CCL2 (formerly MCP-1), are recruited to sites of active neovascularization in close proximity to blood vessels via ANG2/TIE2 interactions (Mazzieri et al, 2011). Following migration into ischemic muscle, tissue-resident TEMs are likely to be further modulated within the hypoxic microenvironment, where they may promote endothelial cell survival and vascular remodelling. The regulation of TEM function by hypoxia-driven pathways in CLI is also supported by recent evidence that F4/80^+^ macrophages in PHD2^+/−^ mice are already skewed to an ‘M2-type’ phenotype, have higher TIE2 expression, and induce greater collateral vessel growth following induction of HLI (Takeda et al, 2011). In the developing embryo, macrophages expressing TIE2 support the formation of blood vessels by physically promoting fusion of sprouting endothelial tips cells via direct cell-to-cell contacts, in a non-canonical, VEGF-independent fashion (Fantin et al, 2010). These cells may have a similar role in providing a scaffold and/or paracrine support during vascular maturation within ischemic tissues.

ANG2 is also important in ‘priming’ the vasculature for angiogenesis by inducing pericyte detachment to destabilize the vessels and increase vascular permeability, which (in the presence of VEGF) promotes endothelial tip-cell sprouting. There is, however, conflicting evidence for the role of ANG2 in ischemia-induced vascular remodelling as its over-expression in endothelial cells has been shown to impair revascularization (Reiss et al, 2007). Our studies reveal the presence of an angiogenic drive in the circulation of patients with CLI, with raised levels of VEGF and ANG2. The latter may be responsible for the upregulation of TIE2 expression that we have measured in circulating monocytes in CLI patients. There is also evidence from other studies that ANG2 enhances the expression of proangiogenic genes (*e.g*. matrix metalloproteinase9, MMP9) or ‘M2’ markers on monocytes (Coffelt et al, 2010).

We have shown that TEMs have proangiogenic activity when delivered into ischemic tissues, hence these cells may deserve further investigation as a potential candidate for cell therapy to promote neovascularization in CLI. Their relatively low abundance in the circulation is, however, an obstacle to their clinical use. This may be overcome in a number of ways. For example, mononuclear cells can be primed with cartilage oligomeric matrix protein-ANG1 (COMP-ANG1) prior to delivery; this was shown to upregulate TIE2 expression on monocytes and to stimulate neovascularization in the ischemic hindlimb (Kim et al, 2009). BMNCs can also be differentiated into TIE2+CD11b+ myeloid cells *in vitro* and used to successfully treat the ischemic hindlimbs of diabetic mice (Jeong et al, 2009). Moreover, TEM-like proangiogenic monocytes/macrophages generated from human embryonic stem cells can also stimulate remodelling and vessel maturation (Klimchenko et al, 2011) and may be used as an alternative and abundant source of these cells.

## MATERIALS AND METHODS

An expanded description of the methods used is available in the Supporting Information.

### Characteristics of patients and controls

Patients with CLI, matched controls and young healthy controls were recruited into this study. Patients with chronic renal failure, a history of malignancy or those taking steroids were excluded. Matched controls were volunteers without clinical evidence of peripheral vascular disease. Venous blood was taken from the antecubital fossa prior to and 12-weeks after intervention to treat CLI (angioplasty, bypass or amputation). Muscle biopsy specimens were taken from patients undergoing lower limb amputation surgery; the normoxic muscle biopsy was taken from the proximal, healthy portion of the leg and the ischemic biopsy from muscle at the distal part of the amputated portion of the limb.

### Quantification of TEMs in blood and muscle

TEMs were quantified in blood and muscle from CLI patients and after induction of HLI in mice (see Supporting Information). Human and murine blood and muscle samples were analysed using flow cytometry. Human monocytes, identified as lineage (CD3,CD56,CD19) negative cells that expressed CD14, were quantified for their expression of TIE2. Murine monocytes were identified as lineage (CD3,CD19,Ly6G,NK1.1) negative, CD11b^+^CD115^+^ cells and quantified for their expression of TIE2.

Human healthy and ischemic muscle biopsies and murine crural muscle samples were digested by incubation in collagenase IV, DNAse and hyaluronidase at 37°C for 30 min followed by trituration and filtration through a 70 µM nylon mesh. Cell suspensions were washed and blocked with the appropriate blocking antibodies prior to staining. Cells obtained from human muscle were fixed with 2% paraformaldehyde and permeabilized with saponin (Perm/wash buffer, BD Biosciences) for intracellular staining of CD68. Human macrophages were identified as lineage negative CD45^+^CD68^+^ cells and quantified for TIE2 expression. Murine macrophages were identified as lineage negative CD45^+^CD11b^+^F4/80^+^ cells and quantified for TIE2 expression.

Intracellular phosphorylation assays were carried out on PBMCs. PBMCs were isolated from whole blood obtained from CLI patients using Ficoll-Paque Plus (GE Healthcare), and stimulated with 30 ng/mL ANG1 oligomers or 300 ng/mL ANG2 (R&D Systems) for 5 min at 37°C. Cells were fixed with 2% paraformaldehyde, permeabilized (Perm buffer IV, BD Biosciences) and phosphorylated TIE2, ERK and AKT were measured in TEMs and TIE2^−^ monocytes using flow cytometry.

Flow cytometric data was analysed by FlowJo (Tree Star Inc., USA) and histograms for phosphorylation studies produced using Cytobank (Cytobank Inc., USA) software. For more details see Supporting Information.

### Isolation of TEMS

Human PBMCs were isolated from 100 mLs of venous blood by Ficoll-Paque. Monocytes were enriched from the PBMCs by immunomagnetic selection using anti-CD14 microbeads (CliniMACS, Miltenyi Biotec). TIE2^+^ and TIE2^−^ monocytes (identified according to the panel of antibodies used above) were then isolated by FACS-sorting (Aria II, BD Biosciences) ensuring purities of greater than 95%. Expression of *TIE2* by TEMs was confirmed using RT-PCR. For more details see Supporting Information.

**The paper explained**

PROBLEM:

Peripheral arterial disease can cause a severe restriction to blood flow leading to critical limb ischemia (CLI), which manifests as a constant and intractable pain, often with ulceration or gangrene. In a third of cases, the limb is not suitable for conventional treatments (surgery or angioplasty), necessitating amputation. Proangiogenic cell therapies, aimed at stimulating new blood vessel growth in the limb, have been used in these ‘no option’ patients for limb salvage but with disappointing results. There is controversy as to which cell types are key for promoting therapeutic neovascularization. Monocytes, known to have a role in both angiogenesis and arteriogenesis, are one of the candidates. We investigated whether a subset of monocytes that express TIE2 (TIE2-expressing monocytes, TEMs) and are pivotal to neovascularization in tumours may also have a role in the revascularization of the critically ischemic limb.

RESULTS:

This is the first study to show that TEMs are increased both in the circulation and muscle of patients with CLI. TEM numbers were also raised in mice following induction of hindlimb ischemia (HLI). TEMs isolated from CLI patients had greater proangiogenic activity compared with TIE2-negative monocytes *in vitro*. Conditional silencing of *Tie2* in TEMs halved the rate of revascularization following induction of HLI, whereas delivery of murine macrophages overexpressing TIE2 or human TEMs isolated from CLI patients rescued limb ischemia and prevented limb loss.

IMPACT:

Our results show that TEMs have the potential to improve revascularization of the ischemic limb and may thus represent a novel cell therapy vehicle for promoting limb salvage in CLI. Delivering a highly proangiogenic subset of monocytes, such as TEMs, might be more fruitful in treating CLI than using whole monocytes or mixed populations of mononuclear cells.

### Assessment of the proangiogenic potential of human TEMs

Human umbilical vein endothelial cells (HUVECs, 4 × 10^3^) were co-cultured with FACS-sorted TIE2^+^ or TIE2^−^ monocytes (2 × 10^3^) on µ-slide angiogenesis plates (Ibidi, Germany) that had been coated with 10 µL per well of growth-factor reduced Matrigel Basement Membrane Matrix (BD Biosciences). Cells were incubated for 18 h at 37°C and 5% CO_2_ and endothelial tubules photographed under phase-contrast microscopy. Image-analysis software (Image-Pro Plus, Media Cybernetics) was used to quantify tubule length and area. Each experiment was carried out in triplicate. For more details see Supporting Information.

TEMs (5 × 10^5^), isolated from CLI patients, were injected into the adductor muscles of nude, athymic mice 24 h after induction of HLI and limb salvage (compared with TIE2^−^ monocytes and vehicle control injections) was recorded using paw auto-amputation as the endpoint.

### Measurement of circulating factors in patients with CLI and controls

Plasma samples, collected from patients with CLI and matched controls, were analysed for a panel of angiogenic and inflammatory factors using SearchLight multiplex analysis array (Aushon Biosystems, USA) and quantikine ELISA kits (R&D systems) following the manufacturer's instructions.

### Recovery of the ischemic hindlimb after *Tie2* silencing and enforced expression of *Tie2* in murine monocytes/macrophages

To knockdown *Tie2* in TEMs, we used a previously described inducible LV-based platform (Mazzieri et al, 2011). Following BM reconstruction of lethally irradiated mice with transduced/transgenic cells, TIE2 expression was conditionally silenced specifically in mature hematopoietic cells using alternate daily doxycycline injections throughout the experiment. HLI was induced in *Tie2* knockdown and *Luciferase* control mice and paw perfusion was measured by laser Doppler. Gastrocnemius muscle specimens were harvested at the end of the experiment and analysed for capillary:fibre ratio. For more details, see Supporting Information.

To determine whether TEMs induce revascularization of the ischemic hindlimb, BMDMs were engineered to overexpress TIE2 using a *Pgk*-*Tie2* LV. BM cells were obtained by flushing the femurs of mice, plated and cultured with M-CSF for 5 days to allow monocytic differentiation. These cells were then transduced with *Pgk*-*Tie2* LVs as described previously (Amendola et al, 2009).

### Statistics

Data were analysed with SPSS version 20 (IBM Corp.) and GraphPad Prism version 5 (GraphPad Inc.). Statistical analyses were carried out using Fisher's exact test, Mann-Whitney U test, paired *t*-test and one-way or two-way ANOVA as appropriate. Data from replicate experiments are represented as mean ± SEM. A two-tailed P value of less than 0.05 was considered statistically significant.

### Study approval

The clinical study protocols were approved by the local research ethics committee at Guy's & St Thomas' NHS Foundation Trust and registered on the UK Clinical Research Network portfolio. All subjects provided informed written consent prior to their participation in the studies. All animal studies were performed under (i) the UK Animals (Scientific Procedures) Act 1986 following approval by the local ethics committee and (ii) the Animal Care and Use Committee of the San Raffaele Scientific Institute (IACUC 324, 335, 446, 447).

## Author contributions

ASP, SN, DB, RQA, JH, KM and OTL designed and performed *in vitro* and *in vivo* experiments. ASP, SN, DB and SPG designed and performed animal studies. SE taught, supervised and provided expertise with the murine model of HLI. RS, AI, MW, PS, LGG and LN provided intellectual input into the cellular and animal studies. MDP designed and supervised *Tie2* knockdown and Tie2-BMDM delivery studies. ASP, AS, MDP and BM provided critical input into the overall research direction. ASP, AS, MDP and BM wrote the paper with input from all co-authors who read, edited and approved the final copy of the manuscript.

## Supplementary material

As a service to our authors and readers, this journal provides supporting information supplied by the authors. Such materials are peer reviewed and may be re-organized for online delivery, but are not copy-edited or typeset. Technical support issues arising from supporting information (other than missing files) should be addressed to the authors.
