# Supplementary material for: TIE2-expressing monocytes/macrophages regulate revascularization of the ischemic limb
Source: EMBO Mol Med. 2013 May 7;5(6):858–69. doi: 10.1002/emmm.201302752 (PMC3779448; doi:10.1002/emmm.201302752)
Supplement: Supplementary file 1 [file emmm0005-0858-SD1.pdf]

TIE2-expressing monocytes/macrophages regulate revascularization of the ischemic limb

Ashish S. Patel, Alberto Smith, Silvia Nucera, Daniela Biziato, Prakash Saha, Rizwan Q. Attia, Julia Humphries, Katherine Mattock, Steven P. Grover, Oliver T. Lyons, Luca G. Guidotti, Richard Siow, Aleksandar Ivetic, Stuart Egginton, Matthew Waltham, Luigi Naldini, Michele De Palma, Bijan Modarai

Corresponding author: Bijan Modarai, King's College London, BHF Centre of Research Excellence & NIHR Biomedical Research Centre at King's Health Partners

Review timeline:

Submission date:	15 March 2013
Accepted:	22 March 2013

Editor: Céline Carret

Transaction Report:

Please note that the manuscript was previously reviewed at another journal and the reports were taken into account in the decision making process at EMBO Molecular Medicine. Since the original reviews are not subject to EMBO's transparent review process policy, the reports and author response cannot be published.
