# Supplementary material for: TIE2-expressing monocytes/macrophages regulate revascularization of the ischemic limb
Source: EMBO Mol Med. 2013 May 7;5(6):858–69. doi: 10.1002/emmm.201302752 (PMC3779448; doi:10.1002/emmm.201302752)
Supplement: Supplementary file 2 [file emmm0005-0858-SD2.pdf]

SUPPORTING INFORMATION

TIE2-expressing monocytes/macrophages regulate revascularization of the ischemic limb

Ashish S. Patel[1], Alberto Smith[1], Silvia Nucera[2,3], Daniela Biziato[2,3,4], Prakash Saha[1], Rizwan Q. Attia[1], Julia Humphries[1], Katherine Mattock[1], Steven P. Grover[1], Oliver T. Lyons[1], Luca G. Guidotti[5], Richard Siow[6], Aleksandar Ivetic[7], Stuart Egginton[8], Matthew Waltham[1], Luigi Naldini[2,3], Michele De Palma[2,4]; Bijan Modarai[1]

Table of contents

Supporting Methods	2
Figure S1	6
Figure S2	7
Figure S3	8
Table S1	10
References	11

Supporting Methods

Flow cytometry

(i) *Human samples.* Peripheral venous blood, collected in ethylenediaminetetraacetic acid (EDTA) tubes (BD Vacutainer, UK), was incubated with human FcR blocking reagent (Miltenyi Biotec, UK) for 30 minutes on ice prior to incubation with a panel of antibodies for 30 minutes on ice (see Supplementary Table S1 for all antibodies used). Following red blood cell lysis (BD Pharm Lyse) and washing of cells, analysis was carried out on a FACS Canto II flow cytometer (BD Biosciences, UK).

(ii) *Murine samples.* Blood, collected via cardiac puncture into EDTA tubes, was lysed with ammonium chloride and FcR-blocked with rat anti-mouse CD16/CD32 (BD Biosciences) prior to staining.

Immunohistochemical analysis of muscle specimens

Muscle samples were fixed in 4% paraformaldehyde for 1 hour, followed by equilibration in PBS/15% sucrose for 12 hours, PBS/30% sucrose for 48 hours followed by PBS/40% sucrose for 24 hours. Tissues were embedded in optimal cutting temperature (OCT) compound and snap-frozen in liquid-nitrogen cooled isopentane. Sections (5μM) were blocked with 5% fetal calf serum in PBS containing 1% bovine serum albumin (BSA) and 0.1% Triton X-100. For staining, unconjugated antibodies were used with secondary antibodies to reveal binding of unconjugated antibodies (see Supporting Information Table S1 for antibodies used). Images were captured using a camera-mounted (EXi Blue, QImaging, UK) fluorescent microscope (Leitz, Lieca, UK) and analysed using image analysis software (Image-Pro Plus, Media Cybernetics, UK).

In vitro angiogenesis assay

HUVECs (Health Protection Agency Culture Collections) were maintained in endothelial basal medium containing the following: 1 ug ml^{-1} hydrocortisone, 100 u ml^{-1} penicillin, 100 ug ml^{-1} streptomycin sulphate, 250 ng ml^{-1} amphotericin B, 10 ng ml^{-1} recombinant human endothelial growth factor, 3 ng ml^{-1} basic fibroblast growth factor, 3 ug ml^{-1} heparin and 2% fetal calf serum. Prior to seeding for tubule formation, HUVECs (of no more than 8 population doublings) were serum-starved for 6 hours.

Hematopoeitic stem/progenitor cell (HS/PC) isolation, transduction, and transplantation for *Tie2* knockdown in TEMs

Bone marrow (BM) was obtained from the femurs of 6- to 12-week-old FVB/*Pgk*-rtTA-miR-126T transgenic mice and lineage-negative cells (enriched in HS/PCs) isolated using a cell purification kit (StemCell Technologies). Cells were then pre-stimulated for 4–6 hr in serum-free StemSpan medium (StemCell Technologies) containing a cocktail of cytokines (IL-3 [20 ng ml^{-1}], SCF [100 ng ml^{-1}], TPO [100 ng ml^{-1}] and FLT-3L [100 ng ml^{-1}], all from Peprotech, Italy) and 10^6 cells ml^{-1} transduced with amiR-expressing LVs, with a dose equivalent to 10^8 LV transducing units ml^{-1}, for 12 hours. Following transduction, 10^6 cells were infused into the tail vein of lethally irradiated, 4-week–old, female FVB/n mice (radiation dose: 950 cGy split in two doses). Starting 8 weeks after HS/PC transplantation (i.e. 12 weeks of age), mice received intra-peritoneal injections of doxycycline (0.5 mg per mouse) 48 hours prior to induction of ischemia and then every alternate day.

FACS-sorting of myeloid cells from spleen

To confirm *TIE2* knockdown in TEMs, OFP^{+} and OFP^{-} myeloid cells were isolated from spleens of both FVB/amiR(*Tie2*) and FVB/amiR(*Luc*) mice at 4 weeks after HLI induction (i.e. at 12 weeks after bone marrow transplant). Spleens were excised and made into single-cell suspensions by trituration and filtration through a 40µM nylon mesh. Red blood cells were lysed with ammonium chloride. Before sorting, we pooled cells from 3 mice belonging to the same group. All cell suspensions were incubated with rat anti-mouse FcγIII/II receptor (CD16/CD32) blocking antibodies (4 µg/mL, BD Biosciences) together with APC-eFluor780 conjugated rat anti-mouse CD11b monoclonal antibodies (eBiosciences). After antibody staining, the cells were re-suspended in 7-AAD-containing buffer, to exclude nonviable cells from further analyses. OFP was acquired as direct fluorescence in the FL2 channel. Cells were sorted using a MoFlo apparatus (Dako). We sorted 7-$AAD^{-}CD11b^{+}OFP^{+}$ and 7-$AAD^{-}CD11b^{+}OFP^{-}$ from spleens of both FVB/amiR(*Tie2*) and FVB/amiR(*Luc*) (n=3; i.e. 3 biological samples/group and each sample represent a pool of cells from 3 mice). After sorting, purity of the cells was >95%.

Real-time (RT) PCR

Expression of TIE2 in human TEMs

TIE2 expression was confirmed in human TEMs with RT-PCR. Total RNA was extracted from $1x10^{5}$ to $2x10^{5}$ FACS-sorted $TIE2^{+}$ and $TIE2^{-}$ monocytes using the RNeasy Micro kit (Qiagen, UK) and reverse transcribed using the superscript III First-Strand Kit (Invitrogen) according to the manufacturer's instructions. RT-PCR analysis of *TIE2* and *GAPDH* (housekeeper gene) was performed on RNAse-treated cDNA. Analyses were carried out in 3 replicates for each sample, for 40 cycles in a

standard mode on an ABI-PRISM 7900 cycler. SDS 2.2.1 software (Applied Biosystems, UK) was used to analyse the data.

Confirmation of Tie2 knockdown in murine cells

To confirm *Tie2* knockdown in murine TEMs, total RNA was extracted from FACS-sorted OFP^{+} and OFP^{-} myeloid cells using the RNeasy Micro kit and reverse transcribed using the superscript III First-Strand Kit. RT-PCR analyses of *TIE2* and β*2m (*reference gene) were performed using TaqMan probes from Applied Biosystems and carried out in 2 replicates for each sample, for 40 cycles in a standard mode on a Viia7 cycler (Applied Biosystems). SDS 2.2.1 software was used to extract raw data (CT). To determine gene expression, the difference (ΔCT) between the threshold cycle (CT) of *Tie2* mRNA and that of the reference gene was calculated by applying an equal threshold. Unpaired t-test was used to assess differences in *Tie2* expression among samples.

***Tie2*-transduction of bone marrow derived monocytes (BMDMs)**

BM cells were obtained by flushing the femurs of 8-week old male FVB/n mice. Cells were plated in RPMI complete medium (10% FBS; penicillin-streptomycin; glutamine) supplemented with M-CSF (50 ng ml^{-1}), and cultured for 5 days to allow monocytic differentiation. Bone marrow cells were transduced with *Pgk*-*Tie2* LVs as described previously (Amendola *et al.*, 2009) on day 1 post-plating.

Figure S1. ***Identification of monocytes in whole blood.*** **(A)** Typical forward and side scatter pattern of whole, lysed blood showing lymphocyte (L), monocyte (M) and granulocyte (G) populations. **(B)** $CD19^{+}$ B cells (left, red box) $CD56^{+}$ natural killer cells (middle, red box) and $CD3^{+}$ lymphocytes (right, red box) were gated to exclude these cells from analysis; **(C)** $CD14^{+}$ monocytes (red polygon) gated for analysis. **(D)** Doublet exclusion. **(E)** Back-gating of final cell population (red) confirms these as monocytes

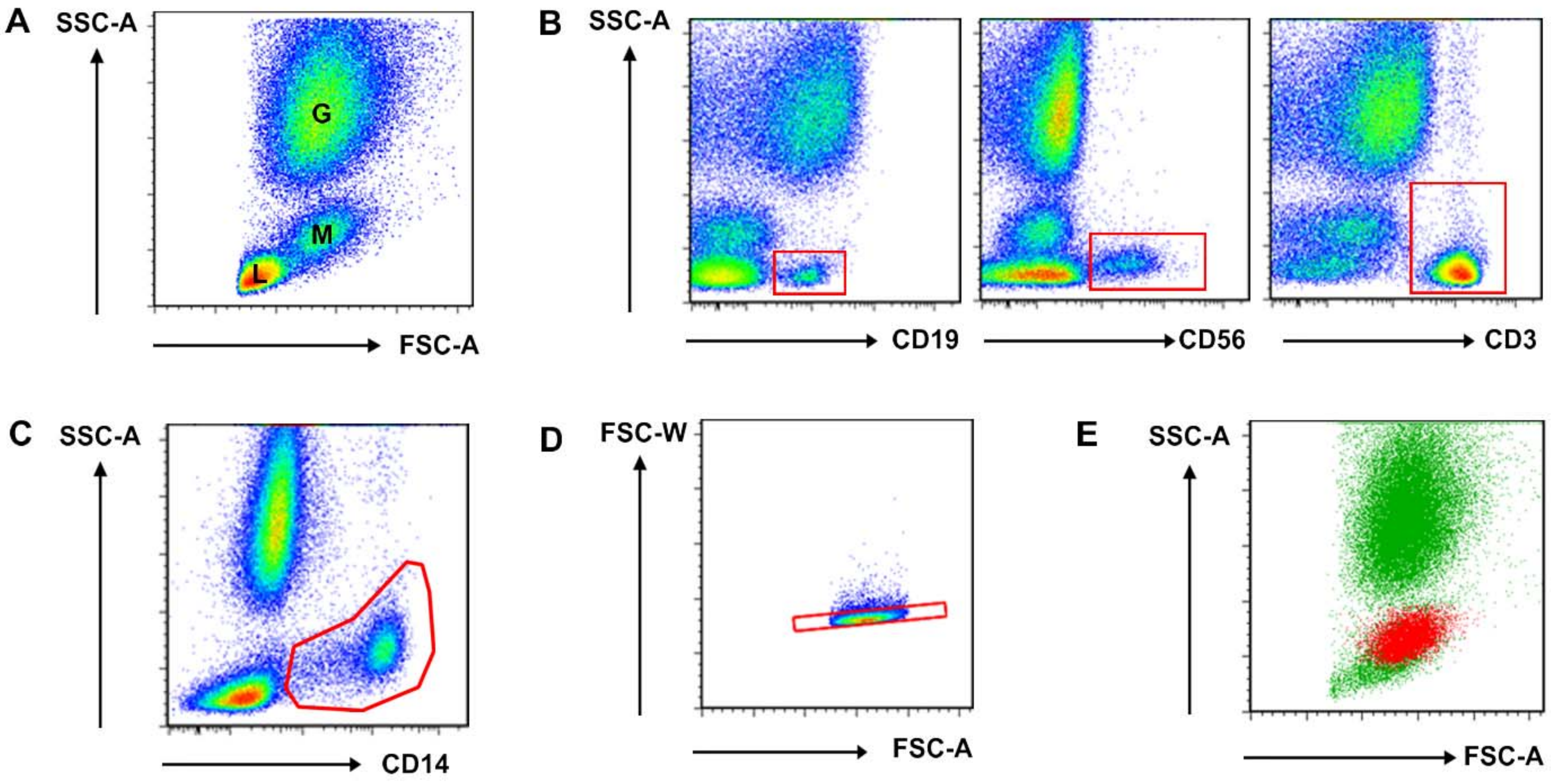

Figure S2. Analysis of TIE2^{+} monocytes and macrophages in blood and digested muscle specimens in the murine hindlimb ischemia (HLI) model. (A) Unilateral paw ischemia following HLI surgery. Laser Doppler flux image shows severe hypoperfusion of the left limb (arrow). **(B)** Analysis of peripheral blood by gating (red boxes) of CD115 positive cells (far left), followed by (CD19, NK1.1, CD3, Ly6G) negative, viable cells (middle left), CD11b^{+} cells (middle right) and TEMs (far right). **(C)** Analysis of murine muscle by gating (red boxes) of CD45 positive cells (far left), followed by (CD19, NK1.1, CD3, Ly6G)$^{-}$ cells (middle left), CD11b^{+} cells (middle right) and F4/80^{+} macrophages (far right). **(D)** Histogram (gated on F4/80^{+} macrophages) showing higher expression of TIE2 in macrophages from ischemic (HLI, red) compared with normoxic (Sham, blue) muscle.

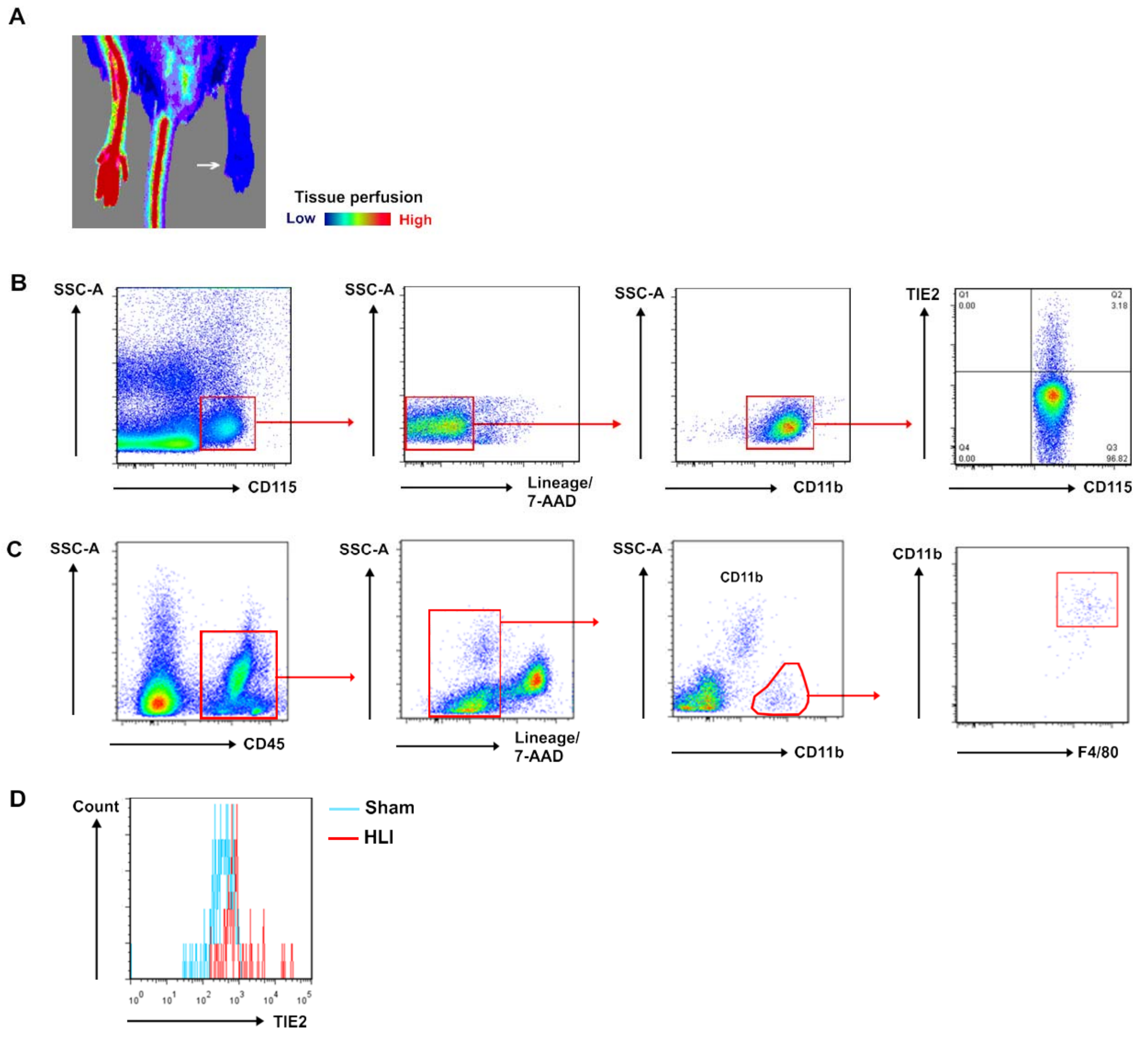

Figure S3. Silencing of *Tie2* expression in TEMs. (A) Flow cytometric analysis showing depletion of lineage positive (B220, Gr1, TER119, CD11b) cells (top row, right dot plot) from bone marrow compared with non-depleted cells (top row, left dot plot). This results in over 10-fold enrichment of $Lin^{-}cKIT^{+}SCA1^{+}$ hemaopoietic stem cells (HSCs, bottom row, right dot plot). **(B)** *In-vitro* administration of 5 mg ml^{-1} doxycycline following LV2 transfection of BM HSCs from *Pgk*-rtTA-miR-126T mice shows induction of OFP expression in both amiR(*Tie2*) and amiR(*Luc*) treated cells to confirm successful transduction. **(C)** Flow cytometric analysis confirms OFP expression in both amiR(*Luc*) (top row) and amiR(*Tie2*) (bottom row) cells following treatment with doxycycline (right column). **(D)** Flow cytometric analysis of peripheral blood following BM transplantation. There is a significant increase in OFP expression in both groups following administration of doxycycline, which is maintained up to day 50 post-transplantation, showing that *Tie2* suppression by the amiR(*Tie2*) cassette remains stable over time. $p<0.0001$ by two-way ANOVA. **$p<0.05$ by one-way ANOVA with post-hoc Bonferroni test. **(E)** No difference in doxycycline-mediated OFP expression in the BM, spleen and peripheral blood after 50 days. p=ns by two-way ANOVA. **(F)** Comparison of proportions of granulocyte, monocyte and B-cells within the bone marrow showing that the hematopoietic compartment is not affected by knockdown of *Tie2*. No difference in composition of these cells within the spleen **(G)** or peripheral blood **(H)** in amiR(*Tie2*) mice compared with amiR(*Luc*) mice 50 days following doxycycline administration. $p>0.05$ for all comparisons by two-tailed Mann Whitney test. n = 8-10 mice per group.

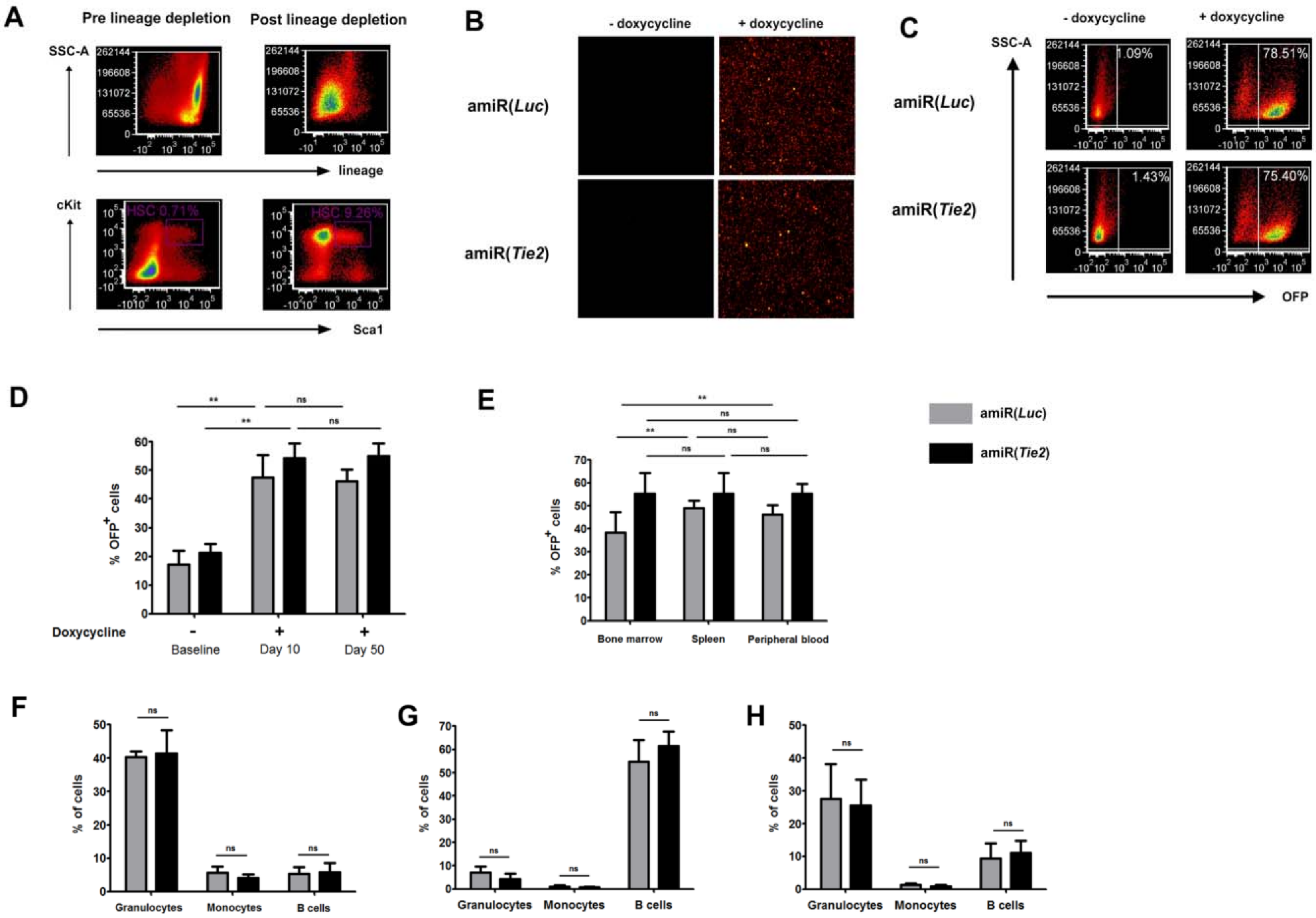
A
Pre lineage depletion
Post lineage depletion
SSC-A
lineage
cKit
Sca1
HSC 0.71%
HSC 9.26%
B
- doxycycline
+ doxycycline
amiR(Luc)
amiR(Tie2)
C
- doxycycline
+ doxycycline
SSC-A
amiR(Luc)
amiR(Tie2)
1.09%
78.51%
1.43%
75.40%
OFP
D
% OFP+ cells
Doxycycline
Baseline
Day 10
Day 50
E
% OFP+ cells
Bone marrow
Spleen
Peripheral blood
amiR(Luc)
amiR(Tie2)
F
% of cells
Granulocytes
Monocytes
B cells
G
% of cells
Granulocytes
Monocytes
B cells
H
% of cells
Granulocytes
Monocytes
B cells

Table S1: List of antibodies used:

Name	Source	Conjugation/secondary antibody
**Human antibodies for FACS analysis**		
CD14	BD Biosciences	FITC
CD45	BD Biosciences	APC
CD68	R&D Systems	FITC
TIE2	R&D Systems	PE
CD3	BD Biosciences	PerCP-Cy5.5 and V450
CD56	BD Biosciences	PerCP-Cy5.5 and V450
CD19	BD Biosciences	PerCP-Cy5.5 and V450
CD16	BD Biosciences	APC
**Human antibodies for immunohistochemistry**		
CD14	Chemicon	Unconjugated/Rabbit anti-mouse Alexa 488
CD68	Abcam	Unconjugated/Rabbit anti-mouse Alexa 488
TIE2	R&D Systems	Unconjugated/Donkey anti-goat Alexa 568
**Murine antibodies for FACS analysis**		
CD115	eBiosciences	APC
CD45	R&D Systems	APC
F4/80	Biolegend	Pe-Cy7
TIE2	eBiosciences	PE
CD3	BD Biosciences	PerCP-Cy5.5
NK1.1	BD Biosciences	PerCP-Cy5.5
CD19	BD Biosciences	PerCP-Cy5.5
Ly6G	BD Biosciences	PerCP-Cy5.5
CD11b	BD Biosciences	APC-Cy7
GR1	Biolegend	Pacific Blue
SCA1	eBiosciences	APC-eFlour780
cKIT	eBiosciences	Pe-Cy7

References

Amendola M, Passerini L, Pucci F, Gentner B, Bacchetta R, Naldini L (2009) Regulated and multiple miRNA and siRNA delivery into primary cells by a lentiviral platform. Mol Ther 17**:** 1039-1052
